# Advance of genetically modified pigs in xeno-transplantation

**DOI:** 10.3389/fcell.2022.1033197

**Published:** 2022-10-10

**Authors:** Jiacheng Deng, Lin Yang, Ziru Wang, Hongsheng Ouyang, Hao Yu, Hongming Yuan, Daxin Pang

**Affiliations:** ^1^ College of Animal Sciences, Jilin University, Changchun, China; ^2^ Chongqing Research Institute, Jilin University, Chongqing, China; ^3^ Chongqing Jitang Biotechnology Research Institute, Chongqing, China

**Keywords:** gene editing, pigs, xeno-transplantation, xenograft donors, xenograft rejection

## Abstract

As the standard of living improves, chronic diseases and end-stage organ failure have been a regular occurrence in human beings. Organ transplantation has become one of the hopes in the fight against chronic diseases and end-stage organ failure. However, organs available for transplantation are far from sufficient to meet the demand, leading to a major organ shortage crisis. To solve this problem, researchers have turned to pigs as their target since pigs have many advantages as xenograft donors. Pigs are considered the ideal organ donor for human xenotransplantation, but direct transplantation of porcine organs to humans faces many obstacles, such as hyperacute rejection, acute humoral xenograft rejection, coagulation dysregulation, inflammatory response, coagulation dysregulation, and endogenous porcine retroviral infection. Many transgenic strategies have been developed to overcome these obstacles. This review provides an overview of current advances in genetically modified pigs for xenotransplantation. Future genetic engineering-based delivery of safe and effective organs and tissues for xenotransplantation remains our goal.

## Introduction

In recent years, the incidence of vital organ failure has increased(Abouna, 2008). Different types of diseases progress to the end stage, and organs are no longer able to meet the most basic needs of the body. Despite the use of drugs and conventional surgery, organ transplantation has become one of the most viable solutions to this problem. To date, more than 106,120 patients have required organ transplants in the United States, while only approximately 40,000 transplants were performed in 2021 (data from URL: https://www.organdonor.gov/statistics-stories/statistics.html). Based on urgent clinical needs, replacing human organs with fully functional animal organs for xenotransplantation therapy is an effective method to address the shortage of donor organs.

Compared with nonhuman primates, pigs have characteristics such as fast reproduction, easy breeding, lower cost, and closer anatomical characteristics and physiological indices to humans, and the use of pigs can avoid the ethical problems caused by the use of nonhuman primate organs (Gao et al., 2021). The use of pigs as donors for pig-to-nonhuman primate (NHP) organ transplantation has become a standard model for preclinical xenotransplantation studies(Klymiuk et al., 2010). However, the clinical application of xenotransplantation still faces many problems: immune rejection of xenotransplantation, abnormal coagulation due to endothelial damage caused by rejection and abnormal growth of transplant donors and biosafety. Gene editing technology has been widely used to solve these problems and prolong the survival rate of organ transplantation. This article reviews the status of xenotransplant organ development and future perspectives.

## Antigens existing in porcine cells that introduce xenograft rejection

The major carbohydrate antigen on porcine vascular endothelial cells has been identified as galactose-α1,3-galactose (α-Gal), to which humans and nonhuman primates have anti-pig antibodies(Cooper et al., 2016). Activation of natural antibodies and the complement cascade mediated by α-Gal (α-1,3-galactosyl) epitopes on the pig cell surface is the main cause of hyperacute rejection (HAR), which leads to severe immune rejection in xenotransplantation (Cowan et al., 2000; Bucher et al., 2005). In 2002, Lai et al. (2002) generated α-1,3-galactosyltransferase knockout pigs, which significantly reduced HAR in pig-to-primate organ transplantation. Subsequently, many research groups have deleted the porcine α-1,3-galactosyltransferase gene and have shown that transplantation of organs from α-1,3-galactosyltransferase knockout (GTKO) pigs significantly prolonged the survival of transplants(Dai et al., 2002; Phelps et al., 2003; Chen et al., 2005; Kuwaki et al., 2005).

Furthermore, previous studies(Chen et al., 2005; Kuwaki et al., 2005; Ezzelarab et al., 2009) have shown that antibody binding to non-Gal antigens and complement activation also lead to xenograft rejection. Acute humoral xenograft rejection (AHXR) caused by non-Gal antibodies and complement activation are obstacles at present. Non-Gal antigens that have been identified to cause AHXR include N-acetylneuraminic acid (Neu5Gc) synthesized by cytidine monophosphate-N-acetylneuraminic acid hydroxylase (CMAH) and Sd^a^ produced by β4GalNT2 glycosyltransferase(Byrne et al., 2015; Wang et al., 2018). Several research groups(Estrada et al., 2015; Martens et al., 2017; Zhang et al., 2018; Tanihara et al., 2021) have developed GGTA1/CMAH/β4GalNT2 knockout pigs, which greatly reduced HAR and AHXR. In 2021, Tanihara’s group(Tanihara et al., 2021) generated GGTA1/CMAH double gene-edited pigs and GGTA1/CMAH/B4GALNT2 triple gene-edited pigs using the CRISPR/Cas9 system, which was the first time that multiple gene-edited pigs had been generated from CRISPR/Cas9-mediated gene-edited zygotes using electroporation. However, there is also some basal reactivity in the TKO (triple knockout) background, leading to poor pig-to-NHP xenotransplantation (Firl and Markmann, 2022).

Martens et al. (2017) revealed SLA class I as an additional target for gene editing in xenotransplantation by screening for human antibody binding using flow cytometric crossmatch (FCXM) in 2017. HLA is a protein complex expressed on human tissue that stimulates the production of new antibodies in allotransplantation. These antibodies can lead to graft failure through hyperacute, acute, or chronic rejection(Ladowski et al., 2021).

In 2014, Reyes et al. (2014) produced piglets lacking the expression of class I SLA proteins, which developed normally. However, class I SLA antigens are critical for viral control in pigs(Ambagala et al., 2000), and class I SLA antigen knockout in pigs still requires long-term evaluation to determine the susceptibility of these animals to infectious diseases and cancer. In 2019, Fischer et al. (2020) produced pigs carrying four gene knockouts of GGTA1, CMAH, B4GALNT2 and either the SLA-I heavy α-chain or light β-chain (B2M), which showed functional knockdown of B2M in animals as well as a lack of SLA-I molecules on the cell surface. However, one group reported negative effects of B2M knockout in mice(Santos et al., 1996). Although the absence of SLA expression is possible, it makes pigs susceptible to infectious complications. A potential alternative effective strategy is to screen key amino acids in SLA by base editor-mediated screening to produce pigs that eliminate cross-reactive binding in the future.

## Human proteins involved in alleviating xenograft rejection

Although knockdown of antigens in pigs helps to reduce graft rejection, there are still other factors that affect graft survival, such as human complement-mediated injury, inflammatory response, and coagulation dysregulation. The expression of human C-reactive proteins (hCRPs) has been reported to prevent damage to pig cells by complement activation(Lin et al., 2009). A number of attempts have been made to deplete or inhibit the complement cascade, generating pigs expressing hCRPs (human C-reactive proteins), hDAF (human decay-accelerating factor, also known as CD55) (Cozzi and White, 1995), hMCP (human membrane cofactor protein, also known as CD46) (Diamond et al., 2001) and hCD59(Fodor et al., 1994). A series of studies have shown that organs from pigs expressing hCRPs effectively resist complement-mediated cytolysis, thereby increasing the survival time after xenotransplantation(Diamond et al., 1996; Ramirez et al., 2000).

However, several other immunological and nonimmunological barriers remain. In 2008, Sprangers et al. (2008) noted that humoral and cellular immune-mediated acute vascular rejection (AVR) mechanisms play key roles in xenotransplantation. The human A20 gene (hA20) is considered to be potentially involved in AVR regulation (Opipari et al., 1990; Daniel et al., 2004; Ferran, 2006). AVR is characterized by endothelial cell (EC) activation and coagulation disorder. In 2009, Oropeza et al. (2009) successfully prepared pigs expressing hA20. The expression of hA20 protects cells against TNF-mediated apoptosis and cell damage caused by inflammation. In addition to A20, haem oxygenase-1 (HO-1) is also a potential factor in the regulation of acute vascular rejection (AVR). HO-1 and its derivatives have anti-apoptotic and anti-inflammatory effects and can resist reactive oxygen species(Nath et al., 1992; Ramackers et al., 2008). In 2011, Petersen et al. (2011) reported hHO-1 gene-modified pigs, and hHO-1 expression was detected in various organs and cells cultured *in vitro*, such as heart, kidney, endothelial and fibroblast cells. Moreover, their results demonstrate that HO-1 plays a protective role in TNF-α-mediated apoptosis(Houser et al., 2004; Shimizu et al., 2005; Shimizu et al., 2008; Shimizu et al., 2012).

It has also been shown that thrombotic microangiopathy occurs in most pig grafts, which may induce the recipient to develop consumptive coagulopathy, leading to graft failure. hTM (human thrombomodulin) is a natural anticoagulant. TM inhibits thrombosis by suppressing direct prothrombinase activity through binding to prothrombinase and enhances its activation of protein C, which is an anticoagulant when activated(Conway, 2012; Yazaki et al., 2012). In 2014, Wuensch et al. (2014) created a genetically modified pig expressing hTM and proved that hTM-expressing pig endothelial cells had anticoagulant properties in a human whole-blood assay. In addition, the biological efficacy of hTM indicated that hTM gene-modified pigs could overcome the coagulation incompatibility in pig-to-primate xenotransplantation.

In addition, the expression of other human coagulation-regulatory proteins (endothelial protein C receptor, tissue factor pathway inhibitor, CD39, CD73) has undergone extensive testing (Lee et al., 2008; Roussel et al., 2008; Petersen et al., 2009; Miwa et al., 2010; Mohiuddin et al., 2014c; Iwase et al., 2014; Mohiuddin et al., 2016). It has been demonstrated that human coagulation proteins greatly minimize coagulation-related problems after xenotransplantation, and coexpression of these coagulation proteins can further improve graft survival (Mohiuddin et al., 2014a; Mohiuddin et al., 2014b; Mohiuddin et al., 2014c). CD47 is a negative regulator of macrophages and is widely expressed in many cells(Oldenborg et al., 2000). The production of CD47 gene-edited pigs is an approach to reduce intrinsic and inflammatory responses and thus improve xenograft survival(Navarro-Alvarez and Yang, 2011). Porcine CD47 does not induce SIRPα tyrosine phosphorylation in human macrophage-like cell lines, and the expression of soluble human CD47-Fc fusion protein induces SIRPα tyrosine phosphorylation, thereby inhibiting phagocytosis of porcine cells by human macrophages. Ide et al. expressed human CD47 in porcine cells and fundamentally demonstrated that it reduced phagocytosis (Ide et al., 2007). Subsequent groups have reported prolonged skin graft survival after the use of human CD47-expressing porcine cells, as well as a substantial protective effect of porcine cell expression of human CD47 on xenografts(Tena et al., 2014; Tena et al., 2017; Chen et al., 2019). Inhibiting the activation of human macrophages through the CD47-SIRP-α signaling pathway is a feasible approach to improve the success rate of xenotransplantation.

## Advance of genetically modified pigs in xenotransplantation

**FIGURE 1:**
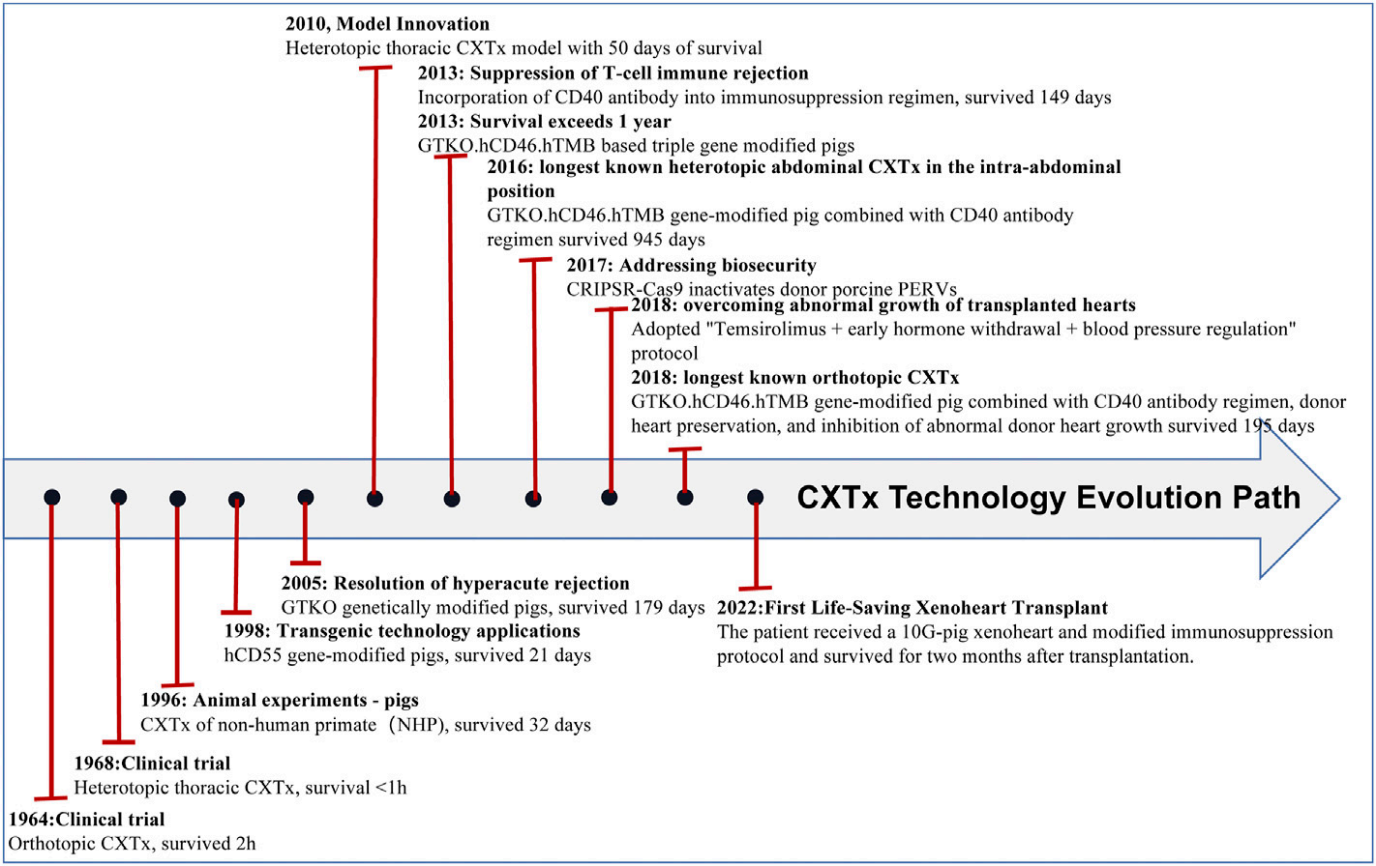
CXTx technology evolution path.

**FIGURE 2:**
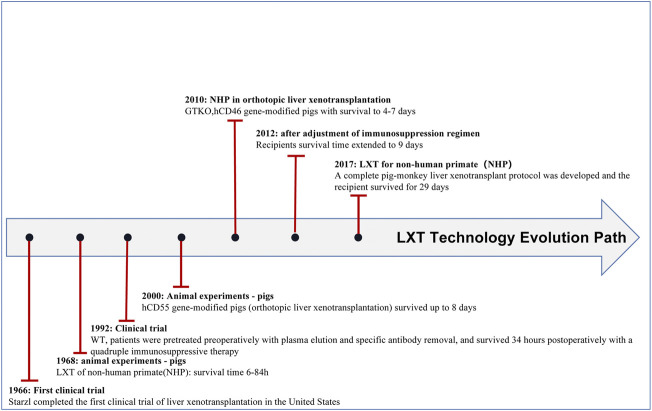
Lxt technology evolution path.

**FIGURE 3:**
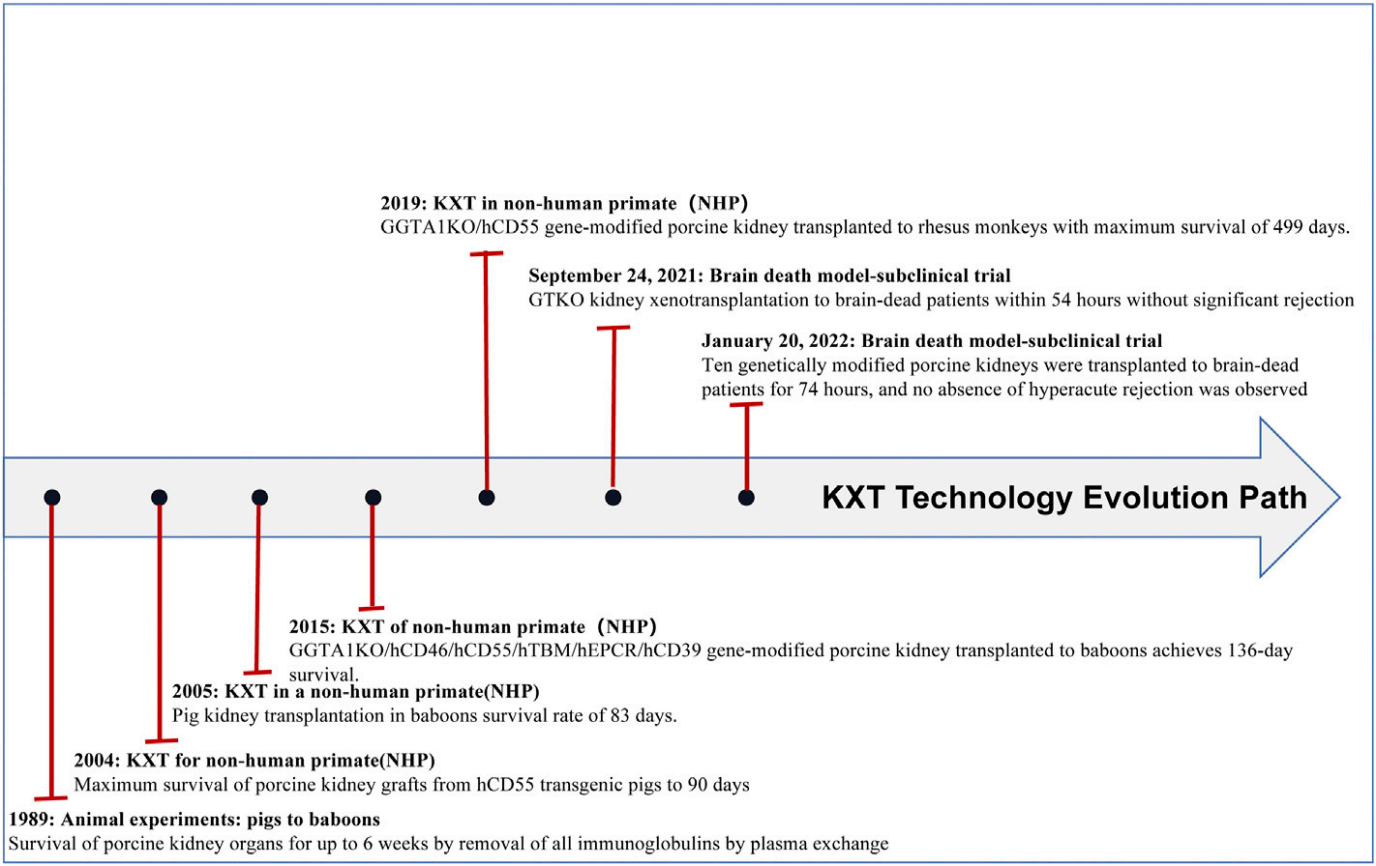
Kxt technology evolution path.

In recent years, the application of gene editing technology has become increasingly common, which has led to prolonged survival of transplanted pig organs in nonhuman primates (NHPs) and a reduced risk of pathogen transfer in organs. Xenotransplantation has made breakthroughs in many fields, especially in heart (see Figure 1 and Table 1), liver (see Figure 2 and Table 2), kidney (see Figure 3 and Table 3), and islet transplantation.

**TABLE 1:** Progress in transgenic porcine heart xenotransplantation.

Year	Recipient	Genetic modifications	Survival	Reason of experiment termination	References
1968	human	WT	<1 h	hyperacute rejection	Adams et al. (2000)
1998	baboon	hCD55	21 d	acute vascular rejection	Baldan et al. (2004)
2005	baboon	GTKO	179 d	thrombotic microangiopathy	Kuwaki et al. (2005)
2010	baboon	GGTA1KO/hCD46	50 d	no signs of infection and active rejection	Bauer et al. (2010)
2013	baboon	GTKO/hCD46/hTBM	499 d	heart xenografts were explanted after rejection and recipient baboons were survived	Mohiuddin et al. (2014a)
2016	baboon	GTKO/hCD46/hTBM	945 d	anti-CD40 significantly prolongs graft survival	Mohiuddin et al. (2016)
2018	baboon	GTKO/hCD46/hTBM	195 d	consistent life-supporting function	Längin et al. (2018)
2022	human	G10	8weeks	multiple organ failure and a porcine virus	Rothblatt, (2022)

**TABLE 2:** Progress in transgenic porcine liver xenotransplantation.

Year	Recipient	Genetic modifications	Survival	Reason of experiment termination	References
1968	baboon	WT	6–84 h	hyperacute rejection	Calne et al. (1968)
1992	human	WT	34 h	hyperacute rejection	Starzl et al. (1999)
2000	baboon	hCD55	8 d	development of sepsis and coagulopathy	Ramirez et al. (2000)
2010	baboon	GTKO/hCD46	4-7 d	thrombocytopenia	Ekser et al. (2010)
2012	baboon	GTKO	9 d	bleeding and enterococcal infection	Kim et al. (2012)
2017	baboon	GTKO	29 d	minimal inflammation	Shah et al. (2017)

**TABLE 3:** Progress in transgenic porcine kidney xenotransplantation.

Year	Recipient	Genetic modifications	Survival	Reason of experiment termination	References
1968	baboon	WT	6–84 h	hyperacute rejection	Calne et al. (1968)
1992	human	WT	34 h	hyperacute rejection	Starzl et al. (1999)
2000	baboon	hCD55	8 d	development of sepsis and coagulopathy	Ramirez et al. (2000)
2010	baboon	GTKO/hCD46	4-7 d	thrombocytopenia	Ekser et al. (2010)

### Advances in heart xenotransplantation

In 1964, Hardy performed the first clinical orthotopic cardiac xenotransplantation (CXTx) when he implanted a chimpanzee’s heart into a 64-year-old male patient who died within 2 h of transplantation(Hardy and Chavez, 1969). Histopathological examination showed that antibody-mediated rejection was the primary cause of the patient’s death(Murthy et al., 2016). In 1968, Donald performed the first clinical heterotopic abdominal CXTx by implanting a wild-type porcine heart into a patient who died of hyperacute rejection (HAR) within minutes after receiving the xenotransplanted heart (Figure 1; Table 1) (Adams et al., 2000), which was intended to confirm the feasibility of human heart transplantation and to provide experience for subsequent human xenotransplantation. In 1998, the Waterworth group(Waterworth et al., 1998) attempted to transplant transgenic porcine hearts to NHPs. They transplanted hCD55 gene-modified pig hearts into baboons, and histological studies showed acute vascular rejection resulting in graft failure (Figure 1; Table 1). Expression of the hCD55 gene extended survival to 21 days and abrogated hyperacute rejection. In 2005, the Kuwaki group(Kuwaki et al., 2005) used alpha1,3-galactosyltransferase knockout pigs as donors for heart transplantation in baboons, which further prevented hyperacute rejection and prolonged survival time to 2–6 months, though xenograft injury due to thrombotic microangiopathy occurred. The transplantation of hearts from galactosyltransferase gene knockout pigs increases graft survival compared to previous studies. In 2010, Bauer’s group(Bauer et al., 2010) performed the first heterotopic thoracic pig-baboon heart transplantation, where the recipient heart could assist the donor heart during rejection episodes, and the recipient eventually survived for 50 days compared to the orthotopic transplant.

An immunosuppressive regimen of co-stimulation blockade *via* anti-CD154 antibodies significantly prolonged cardiac xenograft survival, but many coagulation disorders were observed with the use of anti-CD154 antibodies. In 2013, Mohiuddin’s group(Mohiuddin et al., 2014b) replaced anti-CD154 antibody with anti-CD40 antibody in a GTKO/hCD46 Tg pig-to-baboon heterotopic allograft model, and graft survival was prolonged, with a maximum survival of 146 days. To solve the issue of thrombus formation, GTKO/hCD46 Tg pigs were engineered to express hTBM. In 2013, Mohiuddin’s group transplanted GTKO/hCD46/hTBM pig hearts into baboons, and recipient survival occurred after 1 year(Mohiuddin et al., 2014a). In 2016, the Mohiuddin group(Mohiuddin et al., 2016) achieved recipient survival of 945 days based on a GTKO/hCD46/hTBM modified pig conjugated CD40 antibody regimen. In 2018, the Längin group(Längin et al., 2018) achieved allograft transplantation based on GTKO/hCD46/hTBM combined with nonischemic preservation, continuous perfusion and controlled posttransplant growth of the heart and maintained stable life support function for up to 195 days. Furthermore, on 7 January 2022, Baltimore reported the first-ever life-saving cardiac xenotransplantation. The procedure was successful in extending the patient’s life for 8 weeks. The patient received a 10G-pig xeno-heart (6 human genes knocked-in: CD55, CD46, CD47, human hemeoxygenase-1, human endothelial protein C receptor, hTM; four pig genes knocked-out: Alpha-Gal, Beta4GalNT2, CMAH, growth hormone receptor) and a modified immunosuppression protocol, including costimulation blockade (anti-CD40) maintenance(Rothblatt, 2022). Early published results of posttransplant survival showed that the heart performed very well in the absence of rejection. In the eighth week posttransplant, the patient’s status started to decline, and 2 months after posttransplant, the patient died of multiple organ failure. It is encouraging to see that hyperacute rejection has been defeated. A porcine virus was detected in the transplanted heart and may have been the cause of the patient’s death(Kuehn, 2022).

### Advances in liver xenotransplantation

To address the insufficient supply of living donor livers, liver xenotransplantation is an attractive approach. In 1968, Calne’s group(Calne et al., 1968) performed the first trial of liver xenotransplantation using wild-type pigs as donors, with a maximum survival time of 3.5 days for the recipients, and the longest surviving recipient was treated with an immunosuppressive therapy of glucocorticoids (GC) and azathioprine (AZA). With the application of gene editing, hDAF transgenic pigs with hearts and kidneys that prolong survival and suppress hyperacute rejection have been reported. In 2000, the Ramirez group(Ramirez et al., 2000) first orthotopically transplanted h-DAF gene-modified porcine livers into baboons, which survived up to 8 days postoperatively. The results showed that HAR was abrogated. In 2010, Ekser’s group(Ekser et al., 2010) performed the first orthotopic liver xenotransplantation in baboons using GTKO minipigs transfected with hCD46 as donors, and the baboons survived 4–7 days before dying of abdominal hemorrhage. Baboon survival was prolonged, and hyperacute rejection was further eliminated after transplantation using GTKO. hCD46 pigs as donors compared to hCD46 pigs as donors.

With the elimination of the major obstacle (hyperacute rejection), the current obstacle to the clinical application of liver transplantation is severe thrombocytopenia(Rees et al., 2002; Ekser et al., 2010). Burlak and his colleagues found binding and phagocytosis of human platelets by sinusoidal endothelial cells and Kupffer cells in an *ex vivo* perfusion system. ASGR1 is a receptor expressed by Kupffer cells and hepatocytes that mediates platelet phagocytosis based on the carbohydrate profile of platelets. Paris and his colleagues(Paris et al., 2011) knocked down ASGR1 to reduce ASGR1 expression in asynchronous primary enriched liver sinusoidal endothelial cells (eLSEC) and cripple the ability of primary porcine eLSEC to bind and phagocytose human platelets. Xie’s group(Xie et al., 2021) produced ASGR1-deficient pigs using the CRISPR/Cas9 system. The ASGR1-deficient pigs unexpectedly exhibited mild to moderate liver injury, which has not been reported in humans with ASGR1 variants.

One of the possible approaches to address liver damage resulting from ASGR1 defects in pigs is to screen key amino acid functional loci of proteins at the individual level using base editing techniques. The approach aims to eliminate the liver damage caused by ASGR1 defects in pigs and to effectively alleviate thrombocytopenia in liver xenografts as much as possible.

Shah’s group(Shah et al., 2017) developed a complete pig-to-NHP liver xenotransplantation protocol based on the long-term survival of kidney and heart xenotransplants using GTKO pigs, which do not carry cytomegalovirus, as the organ source. Then, the pigs were supplemented with human clotting factors, followed by applying anti-CD40 monoclonal antibodies to block activation of the recipient costimulatory pathway. For the first time, the protocol allowed for recipient survival following pig-to-primate liver xenotransplantation (LXT) for nearly 1 month. Amino acid and lipid profiles following pig-to-primate liver xenotransplantation suggest that most of the biochemical profiles of porcine liver can be maintained postoperatively in baboons and that supplementation with arginine after LXT may be a potential option to further extend the survival of xenografts(Shah et al., 2019). Based on costimulation blockade with posttransplant administration of human coagulation factors, the team effectively circumvented consumptive coagulopathy and prevented the development of thrombotic microangiopathy (TMA).

Liver xenotransplantation still has a long way to go before undergoing clinical trials, with thrombocytopenia and coagulation dysregulation remaining major hurdles(Li et al., 2021). Gene editing techniques and the combination of tailored immunosuppression and coagulation factor support will likely accelerate the arrival of clinical trials for pig-to-human liver xenotransplantation.

### Advances in kidney xenotransplantation

The rapid development of genome editing technologies such as CRISPR‒Cas9 technology has led to significant progress in kidney transplantation from pigs to NHPs. To date, some groups have achieved more than 6 months of survival in life-supporting pig-to-baboon kidney transplants(Iwase et al., 2017). Recently, the Kim group(Kim et al., 2019) even achieved more than 1 year of survival in life-supporting pig-to-macaque kidney transplants. These recent experiments confirmed the feasibility of kidney transplantation from pigs to NHPs. On 24 September 2021, Robert’s group transplanted a GTKO experimental porcine kidney xenograft into a brain-dead patient, and it functioned immediately after transplantation, urinating and clearing creatinine with no obvious signs of rejection. On 20 January 2022, Porrett’s group(Porrett et al., 2022) performed bilateral native nephrectomies in a human brain-dead decedent. They transplanted a TKO pig kidney with seven additional genetic modifications (ten genetic modifications or 10G-pigs) into a brain-dead patient. The absence of hyperacute rejection (HAR) and the fact that the kidneys remained viable until termination after 74 h suggested that the major barriers to human xenotransplantation had been overcome (Figure 3; Table 3). However, the biopsy revealed thrombotic microangiopathy, which may have been caused by brain death rather than antibody-mediated rejection (AMR). Because the brain death model has many flaws, the next step is expected to be transplanting kidneys from genetically engineered pigs into patients who cannot wait for an allogeneic liver donor.

### Advances in islet xenotransplantation

Pig islet xenotransplantation is a potential approach to patients with type 1 diabetes. In 1994, Groth et al. (1994) performed the first clinical islet xenotransplantation using foetal porcine islet cell-like clusters (ICCs), providing preliminary data for subsequent clinical islet xenotransplantation. There has also been some work in islet xenotransplantation from pigs to NHPs and successful reversal of recipient diabetes and achievement of long-term normoglycemia(Dufrane et al., 2006; Hering et al., 2006; Cardona et al., 2007). Moreover, in some clinical trials(Yang and Yoon, 2015), xenografts were performed using encapsulated neonatal porcine islets, and the grafts were maintained for more than 2 years with a significant reduction in the number of hypoglycemic episodes. In islet xenotransplantation, islet encapsulation and gene editing technologies are currently used to alleviate rejection(Dhanasekaran et al., 2017). Targeted specific removal of porcine endogenous retroviruses from the genomes of porcine cell lines using CRISPR/Cas9 can improve islet xenotransplantation safety. The production of pigs with multiple genetic modifications for xenotransplantation using the targeted specificity of CRISPR/Cas9 has been discussed in this paper and will not be discussed here.

## Discussion

Although the current work has effectively reduced the occurrence of immune rejection, the cross-species infection of pathogens between pigs and humans remains a difficult problem to be solved. This difficulty arises from two aspects: first, overexpression of human genes may increase the risk of human pathogens infecting genetically engineered pigs; second, transplanting pig organs into human bodies may also increase the risk of infection by pig pathogens.

Knock-in of certain human proteins in pigs may enhance the susceptibility of certain viruses to the organism. In engineering genetically modified pigs to overcome immune rejection, human CD46 was introduced into porcine cells to inhibit complement-mediated graft injury(Lu et al., 2019). CD46 not only regulates complement activation and T-cell immunity but is also especially able to control inflammation(Diamond et al., 2001; Astier, 2008; Griffiths et al., 2009). However, CD46 has been shown to be the receptor for measles virus(Okada et al., 1995; Pérez De La Lastra et al., 1999). In addition, hCD55 has been shown to be a receptor for pathogens(Bergelson et al., 1995). Knocking out porcine genomic PERV sequences is a feasible solution to avoid cross-species transmission of PERV and improve the safety of xenotransplantation. Certain groups have performed a large amount of work in this area. Yang et al.(Yang et al., 2015) disrupted all copies of the PERV pol gene in porcine PK-15 at the genome-wide level by using CRISPR/Cas9, reducing the risk of human PERV infection during xenotransplantation by approximately 1000 times. Niu et al. (2017) successfully inactivated all PERV copies in primary pig cell lines using CRISPR/Cas9 and generated PERV-inactivated pigs. Not only are these pigs healthy, but their genome changes are heritable. All of these efforts have effectively addressed the problem of transmission of swine pathogens to humans. In the transplantation of porcine organs into humans, a number of other roseoloviruses may be transmitted and pose a risk in xenografts, such as porcine cytomegalovirus(Denner et al., 2019). Increased viral replication occurs in xenografts during immunosuppression(Mueller et al., 2004). Porcine cytomegalovirus is responsible for a significant reduction in the survival time of transplanted porcine organs. PCMV-negative piglets can be obtained for PCMV elimination through a number of early weaning strategies(Denner, 2022). Eliminating the safety concerns associated with viral infections during xenotransplantation is an essential safety consideration for xenotransplantation.

Furthermore, to further reduce the incidence of immune rejection that is still an issue in current xenotransplants, researchers could try to produce pigs with different genetic modifications using different gene-editing combinations, including knocking in human genes and knocking out pig immunogenicity-related genes(Hinrichs et al., 2021; Yue et al., 2021), to test whether immune rejection is further effectively reduced. Adopting new editing tools is still a good option. Several groups have attempted to use base editors, such as CBE and ABE, to construct better xenograft model pigs(Yuan et al., 2020; Zhu et al., 2022). CRISPR screening of new factors is also a promising option. The emergence of CRISPR genetic screening tools offers hope for screening for new antigenic factors in xenotransplantation. Zhao’s group(Zhao et al., 2020) constructed the first genome-scale CRISPR/Cas9 libraries for screening studies in pigs. A porcine genome-scale CRISPR/Cas9 knockout (PigGeCKO) library was designed, and key host factors promoting JEV infection in porcine cells were identified. It is theoretically feasible to use a porcine genome-scale CRISPR/Cas9 knockout (PigGeCKO) library to identify novel antigens in xenotransplantation.

From the recent first-ever life-saving cardiac xenotransplantation, patients died of multiple organ failure, and organ grafts died from porcine virus infection(Kuehn, 2022). Therefore, with the hope of the eventual implementation of clinical cardiac xenotransplantation, we think it is important to eliminate porcine virus infections to prolong the lifespan of these clinical grafts. The molecular mechanisms associated with rejection involved in pig liver xenotransplantation are more complex than those in cardiac xenotransplantation(Lu et al., 2019). Thrombotic microangiopathy and systemic consumptive coagulopathy are more severe in grafts after liver xenotransplantation than in xenotransplantations of other organs(Zhou et al., 2022). Therefore, addressing thrombotic microangiopathy and systemic consumptive coagulopathy remains a priority for breakthroughs in liver xenotransplantation. In the field of kidney xenotransplantation, which has recently been performed successfully in a brain-dead patient, NYU porcine kidney transplantation is just the beginning. More clinical data are still needed, and the next step may be to initiate a pig kidney transplant trial in patients with end-stage renal failure. More clinical organ xenotransplantation may begin within a few years, with clinical kidney xenotransplantation going first. This is because in the event of a failed transplant, patients could also be put back on dialysis to stay alive(Porrett et al., 2022). In islet xenotransplantation, a current hot spot is the use of cell encapsulation techniques to protect islets from host immune rejection during the initial stages of transplantation. Additionally, there are now some groups trying to transplant porcine islets into different recipient sites(Zhou et al., 2022).
